# The Des Moines Homœopathic Medical Society

**Published:** 1897-11

**Authors:** 


### THE DES MOINES HOMŒOPATHIC MEDICAL SOCIETY.

DES MOINES, IOWA, October 15th, 1897.

EDITOR OF THE HOMŒOPATHIC PHYSICIAN,
1231 Locust Street, Philadelphia, Pa.

DEAR DOCTOR:—At the regular monthly meeting of the Des Moines Homœopathic Medical Society held in this city on September 21st, 1897, the accompanying resolutions were presented and unanimously adopted.

The Secretary was instructed to spread the same upon the minutes, and furnish a copy of the same to the various homœopathic journals for publication.

Very respectfully yours,
WM. WOODBURN,
*Secretary.*

Resolutions unanimously adopted at the regular meeting of the Des Moines Homœopathic Medical Society, September 21st, 1897:

WHEREAS, A report was made at Buffalo upon the application of Dunham College for representation in the Inter-Collegiate Committee of the Institute; and,

WHEREAS, This report, made by a sub-committee of three of its own members, was received by the Inter-Collegiate body without protest or rebuke, in spite of its obviously puerile and malicious character; therefore,

*Resolved,* That this society views with serious alarm and deep mortification the readiness of the Inter-Collegiate Committee, as well as its sub-committee, to give way to petty pique, and abandon itself to cheap and mendacious abuse, instead of maintaining that unprejudiced and judicial attitude which should rightfully be expected of it.

*Resolved,* That the profession, through its National, State, and local societies, should put into practical execution its disapproval of persecution of the younger by the older colleges. This is a matter of far greater reach and importance than the mere question of what the Inter-Collegiate Committee may or may not do in the case of Dunham College. The disgraceful report of its sub-committee is but a single illustration in a single case, of what has been going on for twenty-five years, in countless ways, and to the detriment of deserving colleges. The fact that an unworthy college may be started now and then is no reason—is not even an excuse—for heaping villification upon every new enterprise. It is time that we ceased to hear about the crime of establishing a homœopathic college. It is time to put a stop to this thing of making it all a man's reputation is worth to help man a new enterprise. The older and larger colleges reap to the full the benefits of the general spread of Homœopathy. They have larger classes and greater prestige than they could possibly have had but for the newer and smaller colleges which have aided in upbuilding the cause, and added to the weight of its general standing.

*Resolved*, That this society makes special request of all societies, large and small, all over the country, that they give utterance on this subject, and thus put an end to this unhappy persecution which for twenty-five years has been an ugly and needless hindrance to our progress. If the profession will only voice its sentiments, we shall have an end of the assumption that outside of pre-existing faculties there are none competent to be teachers; the new college in the metropolis will no longer be a "kindergarten;" the new college in the lesser city will no longer be "provincial;" and in estimating the standing of each, malice will give place to merit.

*Resolved*, That a copy of these resolutions be furnished to the medical journals for publication.

